# Neoadjuvant systemic and hyperthermic intraperitoneal chemotherapy combined with cytoreductive surgery for gastric cancer patients with limited peritoneal metastasis: a prospective cohort study

**DOI:** 10.1186/s12885-020-07601-x

**Published:** 2020-11-16

**Authors:** Pengfei Yu, Zeyao Ye, Gaiguo Dai, Yanqiang Zhang, Ling Huang, Yian Du, Xiangdong Cheng

**Affiliations:** Department of Gastric Surgery, Institute of Cancer Research and Basic Medical Sciences of Chinese Academy of Sciences, Cancer Hospital of University of Chinese Academy of Sciences, Zhejiang Cancer Hospital, Hangzhou, 310022 China

**Keywords:** Gastric neoplasm, Peritoneal metastasis, HIPEC, Chemotherapy, Surgery, Prognosis

## Abstract

**Background:**

There is no currently available treatment for peritoneal metastasis of gastric cancer. This phase II study aimed to evaluate the efficacy and safety of neoadjuvant systemic chemotherapy and hyperthermic intraperitoneal chemotherapy (HIPEC) combined with cytoreductive surgery (CRS) for the treatment of these patients.

**Methods:**

Neoadjuvant chemotherapy comprised two cycles of HIPEC and four cycles of S-1 plus paclitaxel. HIPEC was administered intraperitoneally with paclitaxel (75 mg/m^2^). For systemic chemotherapy, paclitaxel was administered intravenously(150 mg/m^2^) on day 1, and S-1 was administered orally(80 mg/m^2^/day)on days 1–14 of a 3-week cycle. Another two cycles of HIPEC and four cycles of S-1 plus paclitaxel were administered after second diagnostic staging laparoscopy or CRS. The primary endpoints were treatment efficiency and safety; the secondary endpoint was 3-year overall survival (OS).

**Results:**

A total of 40 patients were enrolled and 38 patients have been analyzed. Of these, 18 (47.4%) patients received neoadjuvant systemic chemotherapy, HIPEC and CRS (conversion therapy group), while 20 patients received only chemotherapy and HIPEC (palliative chemotherapy group). Median OS was markedly improved in the conversion therapy group (21.1 months, 95% confidence interval [CI] 16.7–25.6 months) in comparison with the palliative chemotherapy group(10.8 months, 95%CI 7.3–14.2 months, *p* = 0.002). After neoadjuvant systemic chemotherapy and HIPEC, a second laparoscopic exploration was performed, and the prognosis of patients with low peritoneal cancer index (PCI) (PCI < 6) was significantly better than that of patients with high PCI (PCI ≥ 6)(20.1 vs.11.3 months, *p* = 0.006).

**Conclusion:**

Neoadjuvant systemic chemotherapy and HIPEC combined with CRS is safe and feasible, and could potentially improve the prognosis of gastric cancer patients with limited peritoneal metastasis. However, further clinical trials are still warranted.

**Trial registration:**

This study has been registered with ClinicalTrials.gov as NCT02549911. Trial registration date: 15/09/2015.

## Background

Gastric cancer(GC) is the fifth most common cancer and the third leading cause of cancer-related deaths in the world [1]. Recurrence and metastasis are the main factors affecting the prognosis of GC patients, and the peritoneum represents the most common site of metastasis and postoperative recurrence [2]. Nearly 20% of patients with GC are diagnosed with peritoneal metastasis preoperatively or intraoperatively, and more than 50% of patients with stage T3 and T4 disease have peritoneal metastasis after radical resection [3]. Once peritoneal metastasis develops, symptoms such as refractory peritoneal effusion, intestinal obstruction and cachexia may occur, which are the major causes of death in patients with advanced GC [4].

Palliative chemotherapy is the main treatment strategy for peritoneal metastasis of gastric cancer, however, the response rate is low with a median survival time less than 6 months [5]. Recent retrospective studies have reported that the primary tumor and peritoneal metastasis can be well controlled in some patients by comprehensive treatment including systemic and local chemotherapy. Thus, cytoreductive surgery (CRS) of the primary tumor and peritoneal metastasis can be achieved and survival time is significantly improved [6, 7].

Hyperthermic intraperitoneal chemotherapy (HIPEC) is a highly concentrated, heated chemotherapy treatment delivered directly to the abdomen [8]. It can not only maintain the high concentration of drugs in the abdominal cavity, but also improve the anti-tumor efficacy of chemotherapy drugs through the thermo-thermal effect [9]. In recent years, HIPEC has been gradually applied in the treatment of peritoneal metastasis of gastrointestinal cancers, and achieved a good therapeutic effect [10, 11].

Therefore, this study aimed to assess the efficacy and safety of neoadjuvant systemic chemotherapy and HIPEC combined with cytoreductive surgery for the treatment of advanced GC with limited peritoneal metastasis, so as to develop a reasonable treatment strategy for these patients.

## Methods

### Study design

The current prospective, single arm, open phase II clinical study was performed in Zhejiang Cancer Hospital, Hangzhou, China, following the Helsinki Declaration as well as the principles of good clinical practice. The study was approved by the ethics review committee of Zhejiang Cancer Hospital(approval number:IRB-2015-170). A written informed consent was obtained from each study participant. The trial was registered with ClinicalTrials.gov (#NCT02549911).

Inclusion criteria were as follows: (1)advanced GC with suspected peritoneal metastasis, including ascites, ovarian metastasis, or omental metastasis; (2)positive peritoneal cytology or peritoneal dissemination with a PCI ≤ 20 confirmed by diagnostic staging laparoscopy (DSL); (3)absence of other distant metastases;(4) no prior treatment (radiotherapy, chemotherapy, targeted therapy, or immunotherapy);(5) 18–75 years of age and Eastern Cooperative Oncology Group (ECOG) performance status of 0–2;(6)normal organ functions, with serum alanine transaminase (ALT) and aspartate transaminase (AST) levels less than two times the upper limits of the normal ranges (ULNs), serum total bilirubin < 1.5 × ULN, serum creatinine < 1.25 × ULN, platelet counts > 75,000/L, absolute granulocyte counts > 1500/L, hemoglobin levels > 90 g/L, and normal electrocardiogram.

Exclusion criteria were as follows: severe intraperitoneal adhesions; presence of a synchronous or metachronous malignancy; pregnant or lactating patients; severe drug hypersensitivity; mental abnormalities; serious respiratory disease; uncontrolled hypertension, diabetes, and cardiovascular disease.

### Treatment

#### HIPEC

HIPEC was performed twice within 72 h after each diagnostic staging laparoscopy(DSL) or CRS using four drainage tubes that were placed in the abdominal cavity. First HIPEC was usually performed within 24 h of DSL or CRS, followed by a second HIPEC at an interval of 24–48 h. Approximately 3 L of heated 0.9% saline supplemented with paclitaxel (75 mg/m^2^) was circulated for 60 min. The heated perfusion solution was infused into the peritoneal cavity at a rate of 400–500 ml/min, which was introduced by an automatic hyperthermia perfusion device (RHL-2000B, Madain Medical Devices Co., Ltd., Jilin, China). The perfusate temperatures in the inflow and outflow tubes were obtained in real time, maintaining the perfusion solution in the peritoneum at43.0 ± 0.5 °C.After HIPEC completion, the abdominal fluid was removed as much as possible [12].

#### Systemic chemotherapy

Preoperative systemic chemotherapy (four cycles of paclitaxel combined with S-1) was administered three weeks after the initial HIPEC, while postoperative systemic chemotherapy (four cycles of paclitaxel combined with S-1) was administered four to six weeks after the second DSL or CRS: S-1 (80, 100 and 120 mg/day for body surface area (BSA) below 1.25 m^2^, between 1.25 and 1.5 m^2^ and above 1.5m^2^, respectively) was administered orally, twice a day for two consecutive weeks followed by a one-week rest, and paclitaxel (150 mg/m^2^) was administered intravenously on day 1.Patients with Her-2 expression received targeted therapy (intravenous trastuzumab at 8 mg/kg on day 1 of the initial cycle, and then 6 mg/kg every three weeks).

#### Surgical treatment

**Fig. 1:**
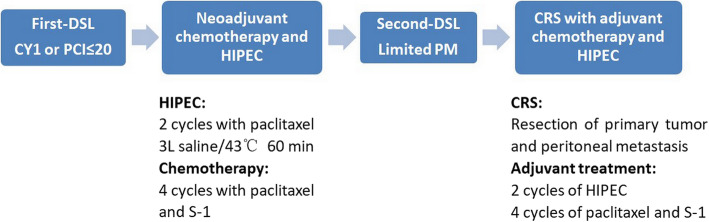
Treatment-schedule for gastric cancer patients with peritoneal metastasis in this study

Four to six weeks after the last preoperative chemotherapy, the patient received a second laparoscopic exploration, if the tumor was well controlled, CRS was performed, including resection of the primary tumor with acceptable margins, lymphadenectomy and peritoneotomy where peritoneal surfaces were involved by tumor [13]. CRS aimed to achieve complete macroscopic cytoreduction; after resection a score estimating the completeness of cytoreduction or CCR was used and defined as: CCR-0 (no residual peritoneal tumor nodules), CCR-1 (residual tumor nodules < 2.5 mm), CCR-2 (residual tumor nodules of 2.5 mm-2.5 cm), and CCR-3 (residual tumor nodules > 2.5 cm or a confluence of unresectable tumor nodules at any site [14]. The treatment schedule is shown in Fig. 1.

### Evaluation

The primary endpoints were treatment efficacy and safety. The secondary endpoint was 2-year overall survival (OS).

The characteristics of the tumor were recorded according to the Japanese gastric cancer classification (3rd edition) [15] and Union for International Cancer Control (UICC) TNM classification (7th edition) [16].Peritoneal metastasis was assessed according to the peritoneal carcinomatosis index (PCI) proposed by Jacquet and Sugarbaker [13]. Histological tumor regression grade (TRG) was assessed as previously described [17]: G1a, complete response; G1b,< 10% residual tumor/tumor bed; G2, 10–50% residual tumor/tumor bed; G3,> 50% residual tumor/tumor bed. A postoperative complication was prospectively defined as any deviation from a predetermined postoperative course within 30 days of surgery and categorized following the Clavien-Dindo severity classification (CDSC) [18]. Adverse events were evaluated using the Common Terminology Criteria for Adverse Events, Version 4.0 (CTCAE v4.0).

### Follow-up

Clinical follow-up was carried out by calling the patients and by accessing outpatient records. It was performed once every 3 months for the first 2 years, followed by once every 6 months for 2–5 years. OS was defined as the time from diagnosis of peritoneal metastasis to last follow up or death. The cutoff date for OS was December 2019.

### Sample size

According to some previous studies [19, 20], the median survival time (MST) of GC patients with peritoneal metastasis was 9–10 months. After neoadjuvant systemic chemotherapy and HIPEC combined with CRS, the MST is estimated to be 18 months. Assuming a two-sided α of 0.05 and 90% statistical power, with an estimated drop-out rate of 20%, the required sample size was estimated to be 40 patients.

### Statistical analysis

Statistical Package for Social Sciences (SPSS ver.19.0 SPSS Inc., Chicago, IL, USA) was employed for data analysis. Student’s t test and the chi-square test were performed for comparing continuous and discrete variables, respectively. OS was determined by the Kaplan-Meier method, and compared by the log-rank test. *P* < 0.05 indicated statistical significance.

## Results

### Patient characteristics

From September 2015 to October 2019, 40 patients were enrolled in this study. Among these, two patients declined chemotherapy and HIPEC. Therefore, a total of 38 patients were finally assessed. There were 18 males and 20 females with the median age of 52 years (range 28–72 years). There were one case with positive peritoneal lavage cytology (CY1), and 37 cases with peritoneal metastasis, including 19 cases with CY1 and 10 cases had ovarian metastasis. After the neoadjuvant treatment, PCI levels in 34 patients (89.5%) were decreased, while increasing in 4 patients. The average PCI score was significantly reduced to 5.8 ± 4.9 from 9.0 ± 5.3 in the whole cohort (*p* = 0.017).A total of 18 (47.4%) patients received neoadjuvant systemic chemotherapy, HIPEC and CRS (conversion therapy group), while 20 patients underwent only chemotherapy and HIPEC (palliative chemotherapy group). Tumor location, differentiation, initial PCI scores, and the levels of tumor markers were comparable in both groups (Table 1).

**Table 1:** Characteristics of the conversion therapy group and the palliative chemotherapy group

Variable	conversion therapy (*n* = 18)	palliative chemotherapy (*n* = 20)	*p*-Value
Gender			0.321
Male	7	11	
Female	11	9	
Median age	49.8(28 ~ 72)	53.5(35 ~ 73)	0.185
Tumor location			
Upper	1	4	
Middle	11	10	
Lower	6	6	0.417
Differentiation			0.758
Well and moderately	9	11	
Poorly	9	9	
Initial DSL			0.321
PCI < 10	11	9	
PCI ≥ 10	7	11	
Second DSL			<0.001
PCI < 6	18	2	
PCI ≥ 6	0	18	
Ascites			0.516
Positive	8	11	
Negative	10	9	
Serum CEA (ng/ml)			0.544
Normal	15	18	
>5	3	2	
Serum CA19–9 (U/ml)			0.914
Normal	12	13	
>39	6	7	
Serum CA125 (U/ml)			0.703
Normal	7	9	
>35	11	11	
Serum CA724 (U/ml)			
Normal	12	15	0.532
>7	6	5	

*DSL* diagnostic staging laparoscopy, *PCI* peritoneal carcinomatosis index

*CEA* carcinoembryonic antigen

### Surgical results

Among the 18 patients who underwent conversion therapy, the second laparoscopic exploration found a PCI of 0 in 6 patients, PCI score of 1–5 in 12 patients (median PCI = 1.5) and CY1 in 3 patients. CCR-0 and CCR-1 were achieved in 15 and 3 patients, respectively. Nine patients were administered total gastrectomy, while 10 patients received combined vascular resections. Of the 20 patients who underwent palliative chemotherapy, the second laparoscopic exploration found PCI score of 5–9 in 13 patients, 10–20 in 7 patients (median PCI = 8.8) and CY1 in 14 patients. Two patients received gastrointestinal bypass, 3 patients had oophorectomy and 7 patients received biopsy of peritoneal metastasis.

A total of 6 (15.8%) patients experienced Clavien-Dindo grade II-III complications, including pneumonia (*n* = 3), anastomotic leakage (*n* = 1) and abdominal abscess (*n* = 2). All of these complications were successfully treated with a conservative procedure.

Histopathological assessment of the resected primary tumors showed that responses to chemotherapy were G1b in 6 patients, G2 in 11 patients and G3 in 1 patient. No patient showed a G1a response (Table 2).

**Table 2:** Surgical data and pathological factors of the conversion therapy group

Variable	*n* = 18
Operating procedure	
Total gastrectomy	9(50.0%)
Distal gastrectomy	9(50.0%)
LN dissection	
D2	14(77.8%)
D2+	4(22.2%)
Combined resection	
Spleen	3(16.7%)
Ovary	7(38.9%)
Pancreas	1(5.6%)
Operation time (min)	223(157–329)
Blood loss (ml)	150(50–800)
Postoperative complications	6(15.8%)
pneumonia	3
anastomotic leakage	1
abdominal abscess	2
Residual tumor	
CCR-0	15(83.3%)
CCR-1	3(16.7%)
yp T grade	
T1	3(16.7%)
T2	5(27.8%)
T3	2(11.1%)
T4a	7(38.9%)
T4b	1(5.6%)
yp N grade	
N0	5(27.8%)
N1	6(33.3)
N2	5(27.8%)
N3	2(11.1%)
Histological response	
G1a	0
G1b	6(33.3%)
G2	11(61.1%)
G3	1(5.6%)

*LN* Lymph node, *CCR* completeness of cytoreduction

### Survival outcomes

The median duration of follow-up in the whole cohort was 19.3 months (range, 5–37 months). The median survival time (MST) of the 38 patients was 15.1 months (95% confidence interval [CI] 11.9–18.4 months); the 1- and 2-year survival rates of the patients were 63.2 and 47.4%, respectively.

**Fig. 2:**
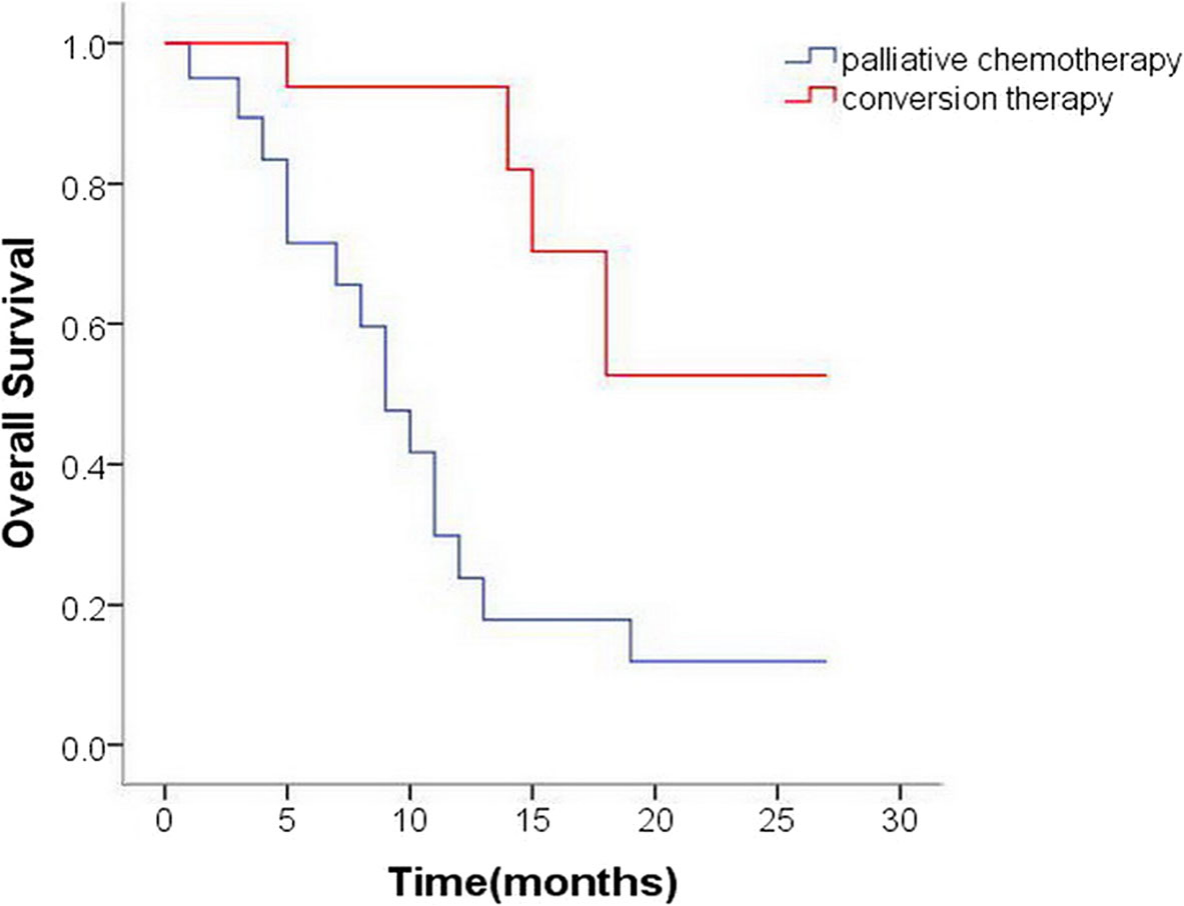
The Kaplan–Meier curve for overall survival of patients who underwent conversion therapy and palliative chemotherapy

**Fig. 3:**
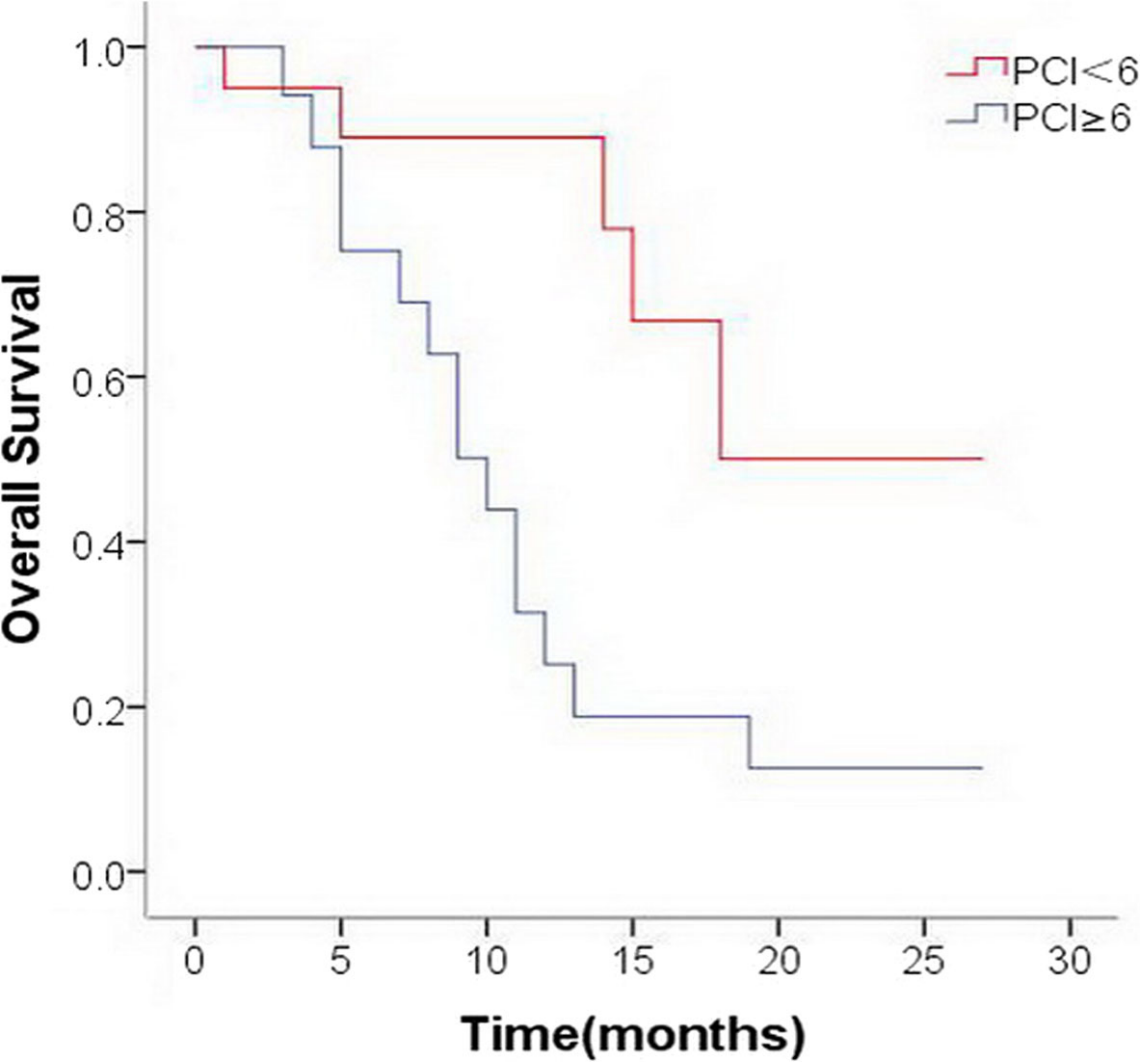
The Kaplan–Meier curve for overall survival of the patients with low PCIs (PCI < 6) and high PCIs (PCI ≥ 6) after neoadjuvant chemotherapy and HIPEC

The median OS in the conversion therapy group was 21.1 months (95%CI 16.7–25.6 months), which was significantly better than that of the palliative chemotherapy group (10.8 months, 95%CI 7.3–14.2 months; *p* = 0.002) (Fig. 2). In the initial diagnostic laparoscopic exploration, patients with low PCI (PCI < 10) had better OS than those with high PCI (PCI ≥ 10), however, the difference was not statistically significant (*p*>0.05). After neoadjuvant systemic chemotherapy and HIPEC treatment, a second laparoscopic exploration was performed, and the prognosis of patients with low PCI (PCI < 6) was significantly better than that of patients with high PCI (PCI ≥ 6)(20.1 vs.11.3 months; *p* = 0.006) (Fig. 3 and Table 3).

**Table 3:** Univariate analysis of predictors for overall survival. (*n* = 38)

Variables	Overall Survival Median	*p*-Value
Age		
≤ 50	16.7	0.478
>50	13.8	
Gender		
Male	12.1	0.120
Female	17.5	
Treatment		
Conversion therapy	21.1	0.002
Palliative chemotherapy	10.8	
Tumor location		
Upper	11.3	0.343
Middle and lower	15.7	
Differentiation		
Poorly	11.6	0.087
Moderately and well	18.1	
Ascites		
Positive	15.3	0.774
Negative	14.5	
Initial DSL		
PCI<10	16.6	0.391
PCI ≥ 10	13.2	
Second DSL		
PCI<6	20.1	0.006
PCI ≥ 6	11.3	
Serum CEA (ng/ml)		
Normal	15.6	0.373
>5	12.0	
Serum CA19–9 (U/ml)		
Normal	15.9	0.359
>39	12.1	
Serum CA125 (U/ml)		
Normal	10.4	0.076
>35	17.6	

*DSL* diagnostic staging laparoscopy, *PCI* peritoneal carcinomatosis index

*CEA* carcinoembryonic antigen

In the conversion therapy group, the 2-year OS rate in chemotherapy-treated patients with TRG of 1 was 100%, whereas that observed for counterparts with TRG of 2–3 was 50%; however, statistical significance was not achieved (*p* = 0.211).

### Treatment toxicity

Chemotherapy combined with HIPEC was well-tolerated in most patients. A total of 10.5% (4/38) of all patients with Her-2 expression received trastuzumab treatment. Grade 3 or 4 adverse events were found in 11 of the 38 patients (28.9%) in the postoperative stage. Among these, the most common hematological toxic effects were leucopenia/neutropenia (15.8%) and thrombocytopenia (5.3%) while the most frequent non-hematological toxic effects were elevation of serum AST levels (7.9%) (Table 4).All the patients suffered from varying degrees of abdominal pain and distension from HIPEC, but these symptoms were adequately controlled by medication.

**Table 4:** Grade 3/4 toxic effects in the two groups (*n* = 38)

Toxic effects	conversion therapy (*n* = 18)	palliative chemotherapy (*n* = 20)
Hematological		
Leucopenia/neutropenia	3(16.7%)	3(15.0%)
Thrombocytopenia	1(5.6%)	1(5.0%)
Non-hematological		
Transaminase elevation	2(11.1%)	1(5.0%)

## Discussion

Peritoneal metastasis has poor prognosis, and is a major cause of death in patients with advanced gastric cancer. In the past, systemic chemotherapy was the main treatment option for these patients [21]. However, the benefit was limited as systemic chemotherapy can be affected by the plasma-peritoneal barrier. HIPEC can maintain a high concentration of drugs in the abdominal cavity and enhance the cytotoxicity of chemotherapeutic drugs against tumor cells under the hyperthermia effect [11]. It has been reported that CRS combined with chemotherapy and HIPEC has the potential to control peritoneal metastasis from GC [6, 7].

There is also some controversy over whether chemotherapy and HIPEC should be performed before CRS. Several clinical studies have demonstrated the clinical efficacy of CRS combined with HIPEC for peritoneal metastasis from GC, although it is associated with high mortality and morbidity [22, 23]. Nevertheless, the clinical significance of CRS plus HIPEC for GC is currently under investigation in the neoadjuvant setting, and this treatment model may be safer and more likely to confer a survival benefit.

**Fig. 4:**
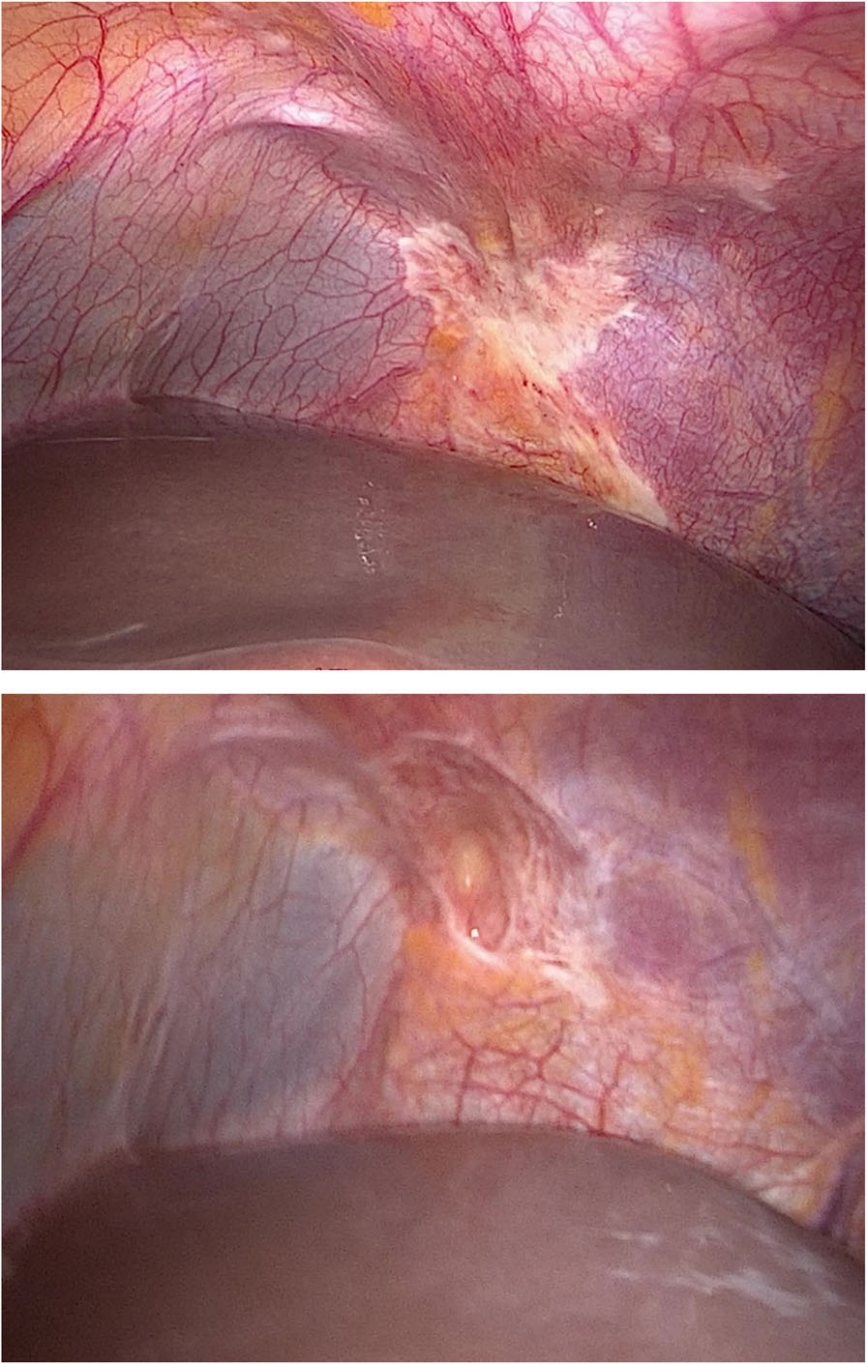
At the first DSL, a flaky metastatic lesion was detected in the left diaphragm, which was confirmed by biopsy as a poorly differentiated adenocarcinoma. After 2 cycles of HIPEC and 4 cycles of paclitaxel and s-1 chemotherapy, the metastasis in the left diaphragm was not obvious, and biopsy suggested scar tissue, with no evidence of tumor

Therefore, a prospective cohort study of neoadjuvant systemic and hyperthermic intraperitoneal chemotherapy combined with cytoreductive surgery was performed to determine the safety and potential survival benefits of conversion therapy for GC patients with limited peritoneal metastasis. This study found that despite the complexity of CRS following neoadjuvant chemotherapy and HIPEC, the incidence of complications was low, and there was no treatment related mortality. After neoadjuvant therapy, the primary tumor and peritoneal metastasis were well controlled (Fig. 4); thus, the scope of surgical resection and the rate of combined visceral resection were reduced. These results are consistent with previous reports [24, 25]. Therefore, CRS after neoadjuvant chemotherapy and HIPEC is safe and feasible for GC with peritoneal metastasis.

A retrospective study assessed 64 GC patients with peritoneal carcinomatosis who received HIPEC combined with systemic chemotherapy; 32 (50%) of these patients who received radical resection showed a median survival of 678 days, which was markedly prolonged compared with that of patients without operation [26]. This prospective study found that after 2 cycles of HIPEC and 4 cycles of neoadjuvant systemic chemotherapy, 18 patients (47.4%) received CRS (conversion therapy). Median OS in the conversion therapy group was 21.1 months, indicating a significant improvement compared with the palliative chemotherapy group. Therefore, after neoadjuvant chemotherapy and HIPEC, peritoneal metastasis could be well-controlled, with survival improved in some patients received complete resection of both primary tumor and peritoneal metastasis.

The evaluation of peritoneal metastasis before conversion therapy is important, and diagnostic staging laparoscopy with PCI assessment is a preferable procedure [27]. It has been proved that PCI is a predictive factor for complete cytoreduction, with the best results obtained when the PCI score is limited [24].In a Chinese study, CRS and HIPEC were administered to GC patients with peritoneal carcinomatosis and/or malignant ascites, and survival analysis indicated that patients with PCI < 20 had significantly better survival than those with PCI > 20 [28]. Another study revealed that patients with incomplete cytoreduction or PCI > 15 were not benefitting from CRS and HIPEC due to a limited survival benefit [29]. The present prospective study demonstrated that after neoadjuvant systemic chemotherapy and HIPEC, PCI scores had been improved in most patients, and the prognosis of patients with low PCI (PCI < 6) was significantly better than that of patients with high PCI (PCI ≥ 6)[20.1 vs.11.3 months, *p* = 0.006].Therefore, after neoadjuvant chemotherapy and HIPEC, only patients with low PCI followed by complete cytoreduction could achieve long-term survival.

The limitations of this study include a relatively small sample size, a single-center design, and short follow up. Therefore, further multi-center large sample studies are warranted to clarify the survival benefits of conversion therapy for GC patients with limited peritoneal metastasis.

Overall, neoadjuvant chemotherapy and HIPEC, combined with cytoreductive surgery, could potentially improve the survival time of GC patients with limited peritoneal metastasis. This procedure is safe and feasible, with only minor complications. However, prospective randomized clinical studies with a large sample size are warranted to validate the results of this study.

## Data Availability

The datasets generated and analysed during the current study are not publicly available due to ethical reasons but are available from the corresponding author on reasonable request.

## Abbreviations

HIPEC: Hyperthermic intraperitoneal chemotherapy
CRS: Cytoreductive surgery
OS: Overall survival
PCI: Peritoneal cancer index
GC: Gastric cancer
DSL: Diagnostic staging laparoscopy
ECOG: Eastern Cooperative Oncology Group
ALT: Alanine transaminase
AST: Aspartate transaminase
ULNs: Upper limits of the normal ranges
BSA: Body surface area
CCR: Completeness of cytoreduction
TRG: Tumor regression grade
CDSC: Clavien-Dindo severity classification
MST: Median survival time
